# Understanding cardiac senescence one cell type at a time

**DOI:** 10.20517/jca.2023.16

**Published:** 2023-05-04

**Authors:** Payel Sen, Priyatansh Gurha

**Affiliations:** 1 Laboratory of Genetics and Genomics, National Institute on Aging, NIH, Baltimore, MD 21224, USA.; 2 Center for Cardiovascular Genetics, Institute of Molecular Medicine and Department of Medicine, University of Texas Health Sciences Center at Houston, Houston, TX 77030, USA.

Cellular senescence, a hallmark of aging, is defined as a state of stress-induced proliferative arrest while maintaining viability and metabolic activity^[1,2]^. Senescence was first characterized *in vitro* by Leonard Hayflick in WI-38 lung fibroblasts after replicative exhaustion and critical telomere shortening^[3]^. Eventually, senescence was observed in multiple cell types including those of cardiovascular origin (cardiomyocytes, cardiac fibroblasts, endothelial cells, *etc.*), and in multiple contexts^[4]^. For example, *acute* senescence was found to be critical for embryogenesis, injury response, and wound healing^[2]^, whereas *chronic* senescence, while initially tumor suppressive, appeared to be associated with a decline in tissue function and even tumor promotion in aging^[5]^.

Acute cellular senescence plays an important physiological role in cardiac development and regeneration, while senescent cells accumulate in the heart with age, leading to an age-related decline in cardiac function^[1,4]^. Accordingly, recent studies have linked cellular senescence and the associated release of inflammatory components to cardiac pathologies. However, the exact role of senescent cells in these diseases is unclear, and in some cases, both adverse and beneficial effects have been reported. Therefore, a more complete understanding of cellular senescence promises new insights into disease pathophysiology and will provide new avenues for disease prevention and treatment.

Regardless of the duration of senescence, its distinguishing molecular hallmarks are persistent DNA damage and profound remodeling of the epigenome and 3D genome that culminates in a distinct transcriptomic and proteomic profile^[6]^. For example, senescent cells express lysosomal beta-galactosidase activity (senescence-associated beta-gal activity or SA-βgal)^[7]^, the cell cycle inhibitor CDKN2A/p16INK4A (hereon p16)^[8]^, and secrete a cocktail of cytokines, chemokines, and matrix remodeling proteins (senescence-associated secretory phenotype or SASP)^[9]^. SASP in the acute senescence context promotes immune clearance of damaged cells, but drives secondary senescence and loss of tissue homeostasis, repair, and regeneration when chronically present. Surprisingly, chronic SASP can also induce cancer stemness^[10]^. All-in-all, SASP is the main culprit in aging and age-related disease, around which several therapeutic strategies called “senotherapies” have been designed.

Elimination of p16-positive senescent cells by a drug-inducible “suicide” gene was shown to improve health and lifespan in both progeroid and naturally aged mice^[11,12]^. Motivated by the mice studies, small molecule drugs called senolytics were designed and became the first class of drugs to target apoptotic elimination of senescent cells in humans^[13]^. Senescent cells heavily depend on anti-apoptotic, pro-survival defenses (called Senescent Cell Anti-Apoptotic Pathway or SCAP). siRNA-mediated knockdown of ~39 SCAP transcripts identified ephrins as key players in the pro-survival pathway. Based on these mechanistic studies, Dasatinib (D, a tyrosine kinase inhibitor) and Quercetin (Q, a natural flavonol) were identified as potent senolytics in part by the ability of Dasatinib to inhibit Ephrin B (EFNB)-dependent signaling and Quercetin’s ability to target phosphatidylinositol 3-kinase (PI3K) and other kinases, and serpines. Interestingly, D+Q showed strong synergistic effects and this combination, to date, remains a popular senolytic agent. In natural or premature aging mouse models, D+Q reduced senescent cell burden and improved cardiac function, carotid vascular reactivity, exercise capacity, and delayed osteoporosis, and loss of intervertebral disk proteoglycans after a single dose^[14]^. In contrast to senolytics, senomorphics (or senostats) do not ablate senescent cells but rather attenuate SASP and its deleterious effects. The first reported senomorphics were rapamycin, metformin, resveratrol, and aspirin^[13]^.

The application of senotherapeutics in cardiovascular aging and disease depends on whether specific cell types display the classic senescence markers. Unlike fibroblasts, cardiomyocyte senescence is somewhat less well characterized, although several markers such as DNA damage, telomere damage/shortening, endoplasmic reticulum stress, mitochondria dysfunction and reactive oxygen species (ROS), contractile dysfunction, hypertrophic growth, and SASP depict senescence features^[4]^. What role cellular senescence of post-mitotic cardiomyocytes has in the pathogenesis of cardiac disease remains to be fully elucidated. Furthermore, cellular senescence in other cardiac cell types, including cardiac progenitor cells (CPCs), epithelial cells, and endothelial cells, and their role in heart disease are poorly defined. Likewise, the cell-type-specific effect of senescence and the intercellular communication required to induce the deleterious effect are of considerable interest. In the case of vascular endothelial cells, for instance, senescent cells have been shown to inhibit angiogenesis, tissue repair and resilience^[1,15,16]^.

In this issue of JCA, Sunderland *et al*. set forth to define the cell-type-specific roles of senescent cells by developing an *in vitro* trans-well assay^[17]^. The authors utilized senescent cardiac progenitor cells (CPCs) or HUVEC cells and evaluated the effect of senescent cells on normal iPSC-derived cardiomyocytes (iPSC-CM) and or HUVEC endothelial cells. Several key findings were noted by the authors.

First, treatment of iPSC-CM with the senescent CPC cells resulted in decreased iPSC-CM survival and cell cycle markers. Additionally, elimination of senescent CPC by D+Q resulted in increased iPSC-CM survival and cell cycle activity, although treatment did not normalize them to wild-type levels. These findings are intriguing in themselves and point to a paracrine signaling mechanism emanating from senescent cells. Indeed, using limited cytokine analysis, the authors identified half a dozen key SASPs induced in senescent CPC and reduced by D+Q treatment. While the exact mechanism of how these cytokines affect iPSC-CM survival or cell cycle activity remains unknown, the result nevertheless validates the detrimental effect of senescent cells on immature cardiomyocytes, such as iPSC-CM.

Second, the authors analyzed the effect of senescent HUVEC and CPC cells on proliferating endothelial cells (HUVEC cells). Endothelial cells are important mediators of tissue homeostasis and repair and involve proliferation, survival, and migration. Using the trans-well assay, the authors demonstrate that senescent cells, regardless of their origin, can reduce HUVEC cell migration and tube formation ability. However, the senescent CPCs have a more profound effect than senescent HUVEC cells on the cell migration and tube formation ability of proliferating HUVEC cells. Additionally, pre-treatment of the senescent cells with senolytic agent D+Q led to a reduction in cytokine levels and an increase in cell survival and migration on the trans-acting cells. It is interesting to note that treatment with senolytics has no effect on the proliferation of HUVEC cells; as a result, the true effect of this treatment on angiogenesis is yet unknown. Nevertheless, these *in vitro* findings suggest a salutary effect of senolytics on endothelial cell migration and thereby imply the ability of these cells to restore cell mobility essential for repair. Overall, the results of this study confirm previous observations and add to the growing literature suggesting the beneficial effect of senolytics on cardiovascular biology^[4,18]^.

While the authors noted several limitations that are self-evident from the *in vitro* studies performed in this manuscript, the results also raise some interesting points that need to be addressed. The authors have developed an *in vitro* system to study the effect of senescent cells and thereby decipher cell-type-specific mechanisms. A comprehensive analysis and characterization of transcriptome and/or proteome of senescent cells and their normal receptive endothelial or cardiac myocytes is warranted to truly define the paracrine and cell-type specific effects. A more detailed analysis of the secretome will be required to identify novel cell-type specific factors that mediate the phenotypic effects. Furthermore, as demonstrated and stated by the authors, the senescent cells were unable to induce the senescence phenotype in the treated normal iPSC-CM or HUVEC cells. The lack of this phenotypic effect, as the authors point out, may be the limitation of the *in vitro* system or the choice of cell type. However, it will be interesting to see if this is also a unique feature of CM, which are inherently non-proliferative and therefore resist induction of senescence without endogenous insult. Finally, the role of DNA damage, a hallmark and sometimes the initiator of the senescent phenotype, was not addressed in this study, nor was the effect of D+Q on DNA damage response.

While the role and function of CPCs in cardiac homeostasis remain unclear, it will be interesting to identify immature cells within the myocardium, which are more amenable to induction of senescence. This will be of great importance in genetic cardiomyopathy, where the primary damage arises from a gene mutation early in life and continues through adulthood. For example, recent studies have shown that the deletion of *Lmna* gene in cardiac fibroblasts leading to dilated cardiomyopathy is associated with induction of cell death and cell senescence marked by induction of DNA damage, senescence-associated beta-galactosidase expression, and induction of SASPs^[19]^. The trans-well assay presented in this manuscript provides an opportunity to study the effect on fibroblast and other cells in these forms of Heart Failure.

While the role and existence of senescent cells are gradually being recognized, the cell-type-specific mechanisms have yet to be elucidated. Finally, to rule out the senescence-independent effect of D+Q, genetic studies with cell-type-specific gene knockout models are needed to consolidate these findings. We surmise that future research will be required to elucidate mechanistic details and develop a “one cell-type at a time” targeting strategy in order to achieve the overall objective of rejuvenation.
